# Enhanced Visible-Light-Driven Photocatalytic Activity by Fe(Ш)-Doped Graphitic C_3_N_4_

**DOI:** 10.3390/molecules27206986

**Published:** 2022-10-17

**Authors:** Zhao Lu, Wulin Song, Minghao Liu

**Affiliations:** 1 Analytical and Testing Center of Huazhong University of Science and Technology, Wuhan 430074, China; 2 State Key Laboratory of Materials Processing and Die & Mould Technology, Huazhong University of Science and Technology, 1037 Luoyu Road, Wuhan 430074, China

**Keywords:** g-C_3_N_4_, Fe(Ш)-doped, photocatalysis, visible light

## Abstract

Fe(Ш)-doped graphitic carbon nitride (Fe(Ш)-CN) photocatalysts with various Fe(Ш) ions content were prepared via ultrasonic method. Detailed physical characterization indicated that Fe(Ш) ions had been successfully doped into the frame of g-C_3_N_4_. The photocatalytic activities were investigated, and methyl orange (MO) and tetracycline hydrochloride (TC) were used as the targeted pollutants. The as-prepared Fe(Ш)-CN materials exhibited higher photocatalytic activities than those of the pure g-C_3_N_4_. Specifically, the degradation rate of 2Fe(Ш)-CN under visible light was 2.06 times higher for MO and 2.65 times higher for TC than that of g-C_3_N_4_. The increased photocatalytic activities of Fe(Ш)-CN were mainly attributed to the enhanced light absorption ability and the rapid separation of photogenerated carriers. Moreover, the importance of active species during the reaction process was also explored, and the results indicated that •O_2_^−^ is the main active species.

## 1. Introduction

Over the past years, numerous efforts have been made to develop highly effective visible light photocatalysts for the treatment of environmental contaminants and solar energy conversion. Numerous metal-based semiconductors have been reported with interesting properties for photocatalytic applications, including TiO_2_, ZnO, WO_3_, SnO_2_, Fe_2_O_3_, BiOX (X = Cl, Br, I), In_2_O_3_ and so on [1,2,3,4,5,6,7,8]. Recently, a kind of free-metal visible-light photocatalyst, graphitic carbon nitrides (g-C_3_N_4_), has received wide attention owing to its abundance, low cost, thermal stability and chemical tenability [9,10,11,12]. It can be synthesized from a simple precursor via polycondensation reactions without any metal involvement [13,14]. However, the photocatalytic efficiency of the pure g-C_3_N_4_ is limited by the poor specific surface area, unsatisfactory visible light utilization and rapid recombination of photogenerated charge carriers. To enhance its photocatalytic performance, tremendous efforts have been made to optimize the nanostructure, surface chemical state and photoelectrical structures, including suitable textural design [15,16], doping with metal and nonmetal elements [17,18,19,20] and constructing heterojunctions with other semiconductors [21,22,23].

As is known to all, anions or/and cations doping is an effective strategy that can expand photoresponse range, decrease the band gap energy and enhance the efficiency of charge separation. For example, Fu et al. [19] reported that O-doping optimized the band structure of g-C_3_N_4_, resulting in narrower bandgap and higher separation efficiency of photo-generated charge carriers. Wang et al. [24] showed that Mn doped g-C_3_N_4_ photocatalysts exhibited high activity towards photocatalytic degradation under visible light irradiation due to the enhancement of photo-induced carrier separation.

Considering the sp2 hybridization of carbon and nitrogen in the g-C_3_N_4_ that forms the p-conjugated graphitic planes, which is similar to the structure of graphite, and the heptazine rings in the g-C_3_N_4_ with six nitrogen lone pair electrons that can serve as electron donors, it has excellent affinity to entrap transition metal ions [25]. In this study, Fe(Ш)-CN was prepared by ultrasonic method using ferric chloride as iron source, and the photocatalytic activities of Fe(Ш)-CN were evaluated by the degradation of MO and TC. The relationship between the photocatalytic activity and the modified property was discussed. The photocatalytic mechanism and the kinetics were also proposed.

## 2. Experimental Section

### 2.1. Synthesis of g-C_3_N_4_

The g-C_3_N_4_ was synthesized by thermal polycondensation of urea. Typically, 10 g of urea was put into a ceramic crucible with a cover, heated from room temperature to 580 °C with a heating rate at 15 °C min^−1^ in a muffle furnace and kept at 580 °C for 2 h. After cooling naturally to room temperature, the resulting pale yellow products were collected and ground into powders. Fe(Ш)-CN was prepared through a simple ultrasonic process. First, 0.2 g of g-C_3_N_4_ was dispersed into 200 mL distilled water and then kept stirring for 4 h. At that point, a certain amount of FeCl_3_ aqueous solution (0.01 g/mL) was quickly added into the solution under continuous vigorous stirring for 1 h, then ultrasonicated for 8 h. Finally, the samples were collected by centrifugation and washed with deionized water six times, followed by drying in a vacuum oven at 60 °C overnight. By varying the dosage of FeCl_3_, a series of Fe(Ш)-CN was synthesized; the mole percentage ratios of Fe(Ш) ions against g-C_3_N_4_ were 0.5%, 1%, 2%, 4% and 8%, labelled as XFe(Ш)-CN, where X is the mole percentage (0.5, 1, 2, 4 and 8) of Fe^3+^ with respect to g-C_3_N_4_.

### 2.2. Characterization

X-ray diffraction (XRD) analysis of all samples was conducted with X-ray diffraction (PANalytical B.V., Almelo, The Netherlands) with a Cu Kα radiation, and their microstructures and morphologies were observed with transmission electron microscopy (TEM, JEOL, JEM-2100F, Japan). EDS mapping was obtained with field transmission electron microscopy (FTEM, FEI, Talos F200X, Czech republic). The molecular structure was investigated using a Fourier transform infrared (FT-IR) spectrometer (Bruker Tensor27, Germany ). X-ray photoelectron spectroscopy (XPS) measurements were done in a VG Multilab2000 spectrometer. UV-Vis diffuse reflectance spectroscopy (UV-Vis DRS, Japan) absorption spectra were recorded with a Shimadzu U-3010 spectrometer using BaSO_4_ as a reference. Photoluminescence (PL) spectra of these powders were obtained on a Jasco FP-6500 with a laser excitation of 325 nm. ESR signals of •O_2_^−^ and •OH with 5, 5-diemthyl-1-pyrroline N-oxide (DMPO) were recorded with a Bruker EMX PLUS spectrometer.

### 2.3. Photocatalytic Properties Characterization

The photocatalytic activities were evaluated by the degradation of MO (20 mg·L^−1^, 50 mL) and TC (20 mg·L^−1^, 100 mL) under visible light irradiation (*λ* > 400 nm). Generally, 50 mg of catalyst was dispersed in the target solution. A 300 W simulated solar Xe arc lamp (CEL-HXF300) with an ultraviolet cutoff filter (400 nm) was used as the light source and positioned 15 cm above the photocatalytic reactor. A liquid trap system was used to eliminate the temperature effect. Firstly, the reaction suspensions were under constant stirring for 30 min in the dark to establish an absorption–desorption equilibrium. Then, the lamp was turned on and 5 mL of solution was taken from the suspensions and centrifuged at a given time interval. Subsequently, the supernatant fluid was extracted immediately. The concentrations of MO and TC were analyzed by UV/Vis spectroscopy. The degradation rate was calculated by *C*/*C*_0_, where *C* was the concentration after irradiation and *C*_0_ was the concentration of the reactant after adsorption–desorption equilibrium. A blank control test without photocatalyst was conducted for reference.

## 3. Results and Discussion

### 3.1. XRD Characterization

**Figure 1:**
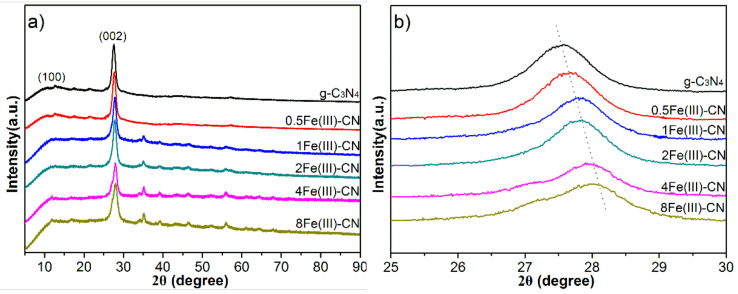
XRD patterns of all samples: 5°–90°(**a**); 25°–30°(**b**).

XRD was used to reveal phase structures of all samples, and the results are shown in Figure 1a. Two distinct diffraction peaks can be found in all samples. The high-intensity peak at 27.5° is ascribed to interplanar stacking of aromatic systems, which is indexed to the (002) peak and corresponded to an interlayer distance of 0.326 nm [13,26]. The peak around 13.1° is indexed as the (100) peak and can be associated with an in-plane structural packing motif [13,26]. As the feeding amount of FeCl_3_ increases, a slight positive shift of the (002) peak can be observed, indicating the shrink in crystal plane (Figure 1b). Since the delocalized pi bond of g-C_3_N_4_ possesses high electron density, which can provide electrons to d orbitals of Fe(Ш) ions, there is a strong mutual attraction between Fe(Ш) ions and g-C_3_N_4_. As a result, the crystal plane spacing (002) becomes smaller with increasing Fe(Ш) ions content. This result implies that the Fe(Ш) ions have been inserted into the interlayer of g-C_3_N_4_ [27]. Meanwhile, there are many small diffraction peaks between 30°–80° in Fe(Ш)-CN samples, which can be indexed to the FeOOH and confirm the presence of FeOOH in the samples [28,29]. Moreover, the peak intensity increases as the amount of FeCl_3_ increases.

### 3.2. TEM Characterization

**Figure 2:**
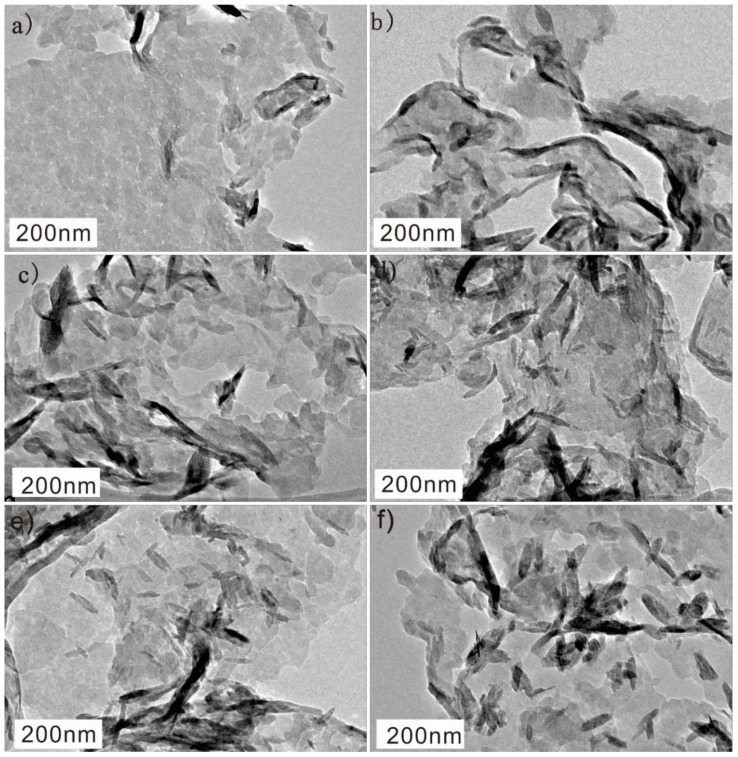
TEM images of all samples: g-C_3_N_4_ (**a**), 0.5Fe(Ш)-CN (**b**), 1Fe(Ш)-CN (**c**), 2Fe(Ш)-CN (**d**), 4Fe(Ш)-CN (**e**) and 8Fe(Ш)-CN (**f**).

The morphologies of all as-prepared samples were investigated via TEM. The TEM image in Figure 2a shows that g-C_3_N_4_ has a two-dimensional (2D) nanosheet structure with plenty of wrinkles. The TEM images of Fe(Ш)-CN samples with varying Fe(Ш) ions content are shown in Figure 2b–f. The 2D sheet-like morphology is well maintained after introducing Fe(Ш) ions. As the Fe(Ш) ions content increases, more and more nanoparticles graft onto the surface of g-C_3_N_4_ nanosheets. The dense particles on the g-C_3_N_4_ can be seen in the 8Fe(Ш)-CN as shown in Figure 2f. Combined with the results of XRD, these nanoparticles are FeOOH species, and high Fe(Ш) ions content might be attributed to the generation of FeOOH species.

**Figure 3:**
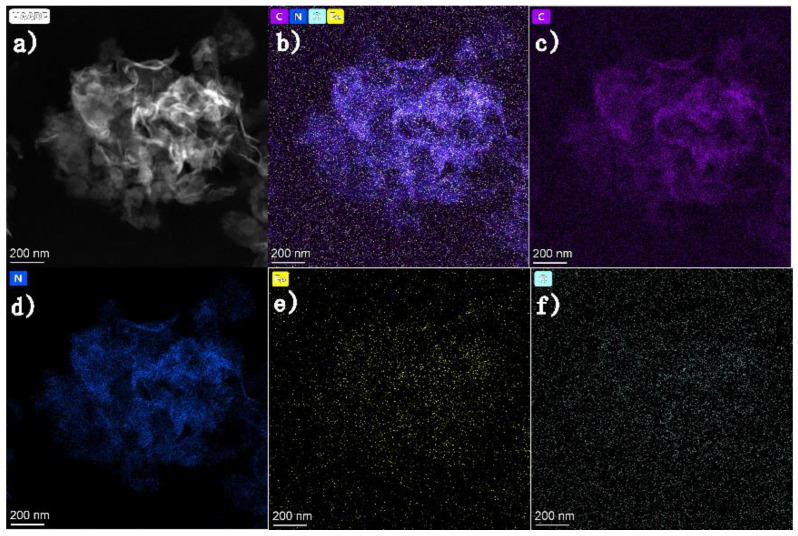
EDS mapping of 2Fe(Ш)-CN: (**a**) HAADF image; (**b**) multi-elemental image of C, N, Fe and O; (**c**) C element map; (**d**) N element map; (**e**) Fe element map; (**f**) O element map.

Furthermore, a detailed chemical analysis was carried out using element mappings as shown in Figure 3. It is clearly seen that C element distribution is generally the same as that of the compositional element N, which is assigned to the g-C_3_N_4_ nanosheets. The distribution of Fe element is distributed across the whole surface of g-C_3_N_4_, demonstrating that Fe(Ш) has been doped into the frame of the g-C_3_N_4_ successfully. Meanwhile, the bright spots in the Fe element map and the O element map point to the FeOOH.

### 3.3. FT-IR Spectra

**Figure 4:**
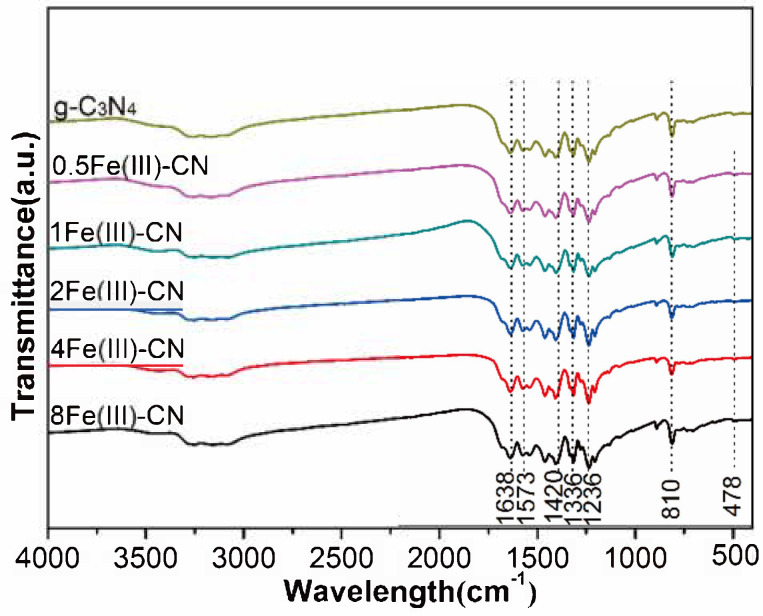
FT-IR spectrum of all samples.

The molecular structure information of all samples was illustrated by FT-IR spectra, as depicted in Figure 4. There are three identical absorption bands in all samples which are assigned to the g-C_3_N_4_ [30,31]. The absorption band at 1638 cm^−1^ corresponds to the typical stretching modes of C-N bond. The prominent bands in the region of 1200–1600 cm^−1^ are related to the typical stretching modes of heptazine heterocyclic ring units. The intense band at 810 cm^−1^ represents the out-of-plane breathing vibration characteristic of triazine units. After the introduction of Fe(Ш) ions, there is an additional absorption band at 478 cm^−1^, which is attributed to the Fe-O characteristic breathing mode [28]. The results accord well with those of XRD and TEM.

### 3.4. Chemical Compositions

**Figure 5:**
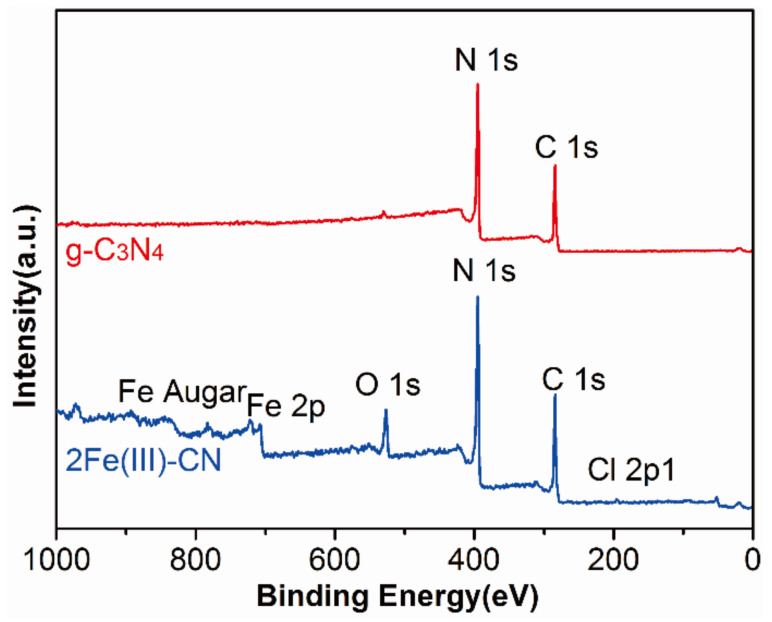
XPS spectra of g-C_3_N_4_ and 2Fe(Ш)-CN samples.

XPS was employed to further identify the chemical states of the g-C_3_N_4_ and 2Fe(Ш)-CN samples. Figure 5 presents the survey scan XPS spectra of the g-C_3_N_4_ and 2Fe(Ш)-CN samples. Five elements including C, N, O, Cl and Fe can be observed in the survey spectra of the 2Fe(Ш)-CN sample.

**Figure 6:**
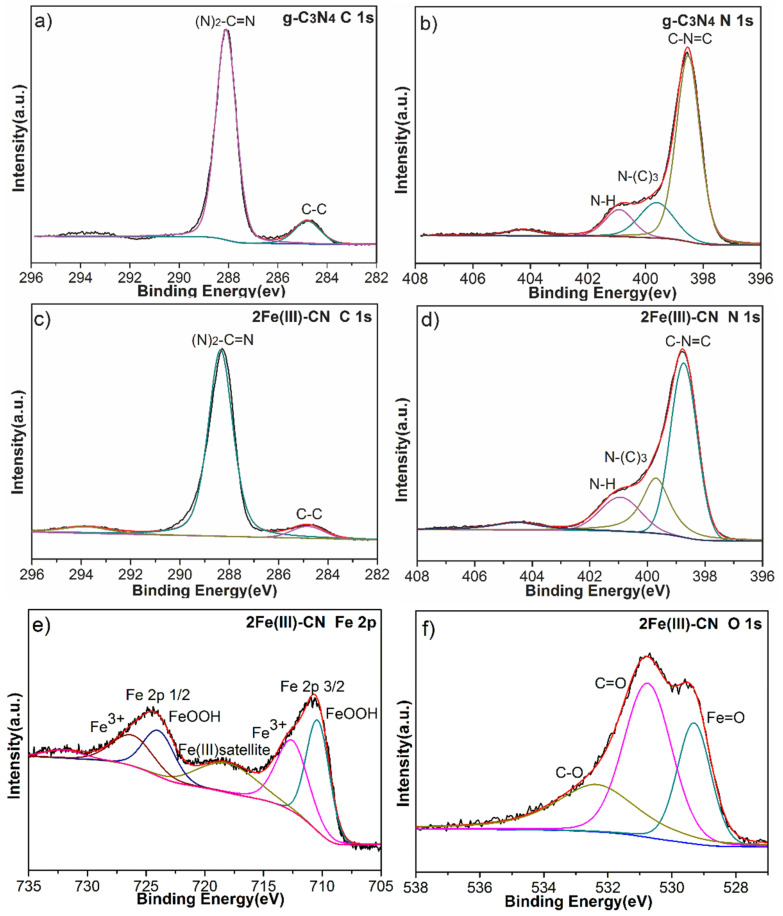
C 1s (**a**) and N 1s (**b**) XPS spectra of g-C_3_N_4_; C 1s (**c**), N 1s (**d**), Fe 2p (**e**) and O 1s (**f**) XPS spectra of 2Fe(Ш)-CN sample.

Figure 6a,c show the high resolution XPS spectra of C 1s region for g-C_3_N_4_ and 2Fe(Ш)-CN, respectively. For the g-C_3_N_4_, the peak at 288.1 eV is assigned to sp2-hybridized carbon in the unit of (N)_2_-C=N [32,33]. After Fe(Ш) ions doping, the peak shifts to a more positive position. The high-resolution N 1s XPS spectrum of g-C_3_N_4_ is shown in Figure 6b. It can be separated into four peaks centered at 398.5 eV, 399.6 eV, 400.9 eV and 404.5 eV, corresponding to the C=N-C, N-(C)_3_, N-H and π-excitations, respectively [32,33]. For the 2Fe(Ш)-CN sample, as shown in Figure 6d, the binding energy of C=N-C (398.7 eV), N-(C)_3_ (399.8 eV) and N-H (401.0 eV) all show a slight increase compared to those of the pure g-C_3_N_4_. The binding energy of both C 1s and N 1s in 2Fe(Ш)-CN sample shift to more positive positions, which is related to the interaction between Fe(Ш) ions and g-C_3_N_4_. Since the delocalized pi bond of g-C_3_N_4_ possesses high electron density, providing electrons to d orbitals of transition metal element Fe, this leads to the decrease in the electron density of C_3_N_4_ and the increase in the binding energy of XPS peaks.

The high-resolution Fe 2p XPS spectrum of 2Fe(Ш)-CN is shown in Figure 6e. Fe 2p3/2 can be deconvoluted into three peaks, with binding energy at 710.4 eV for FeOOH, 712.6 eV for the residual Fe(Ш) salt and 718.1 eV for Fe(Ш) satellite peak. Similarly, Fe 2p1/2 can also be deconvoluted into these three corresponding peaks [28,33,34]. The O 1s peak can be fitted into three peaks with binding energies of 529.3 eV, 530.8 eV and 532.4 eV for 2Fe(Ш)-CN sample (Figure 6f), which can be ascribed to Fe=O, C-O and C=O coordination, respectively [28]. Thus, it can be concluded that Fe(Ш) ions have been successfully incorporated into the frame of g-C_3_N_4_ and some FeOOH nanoparticles have formed in the samples.

### 3.5. UV-Visible Diffuse Reflection Spectra

**Figure 7:**
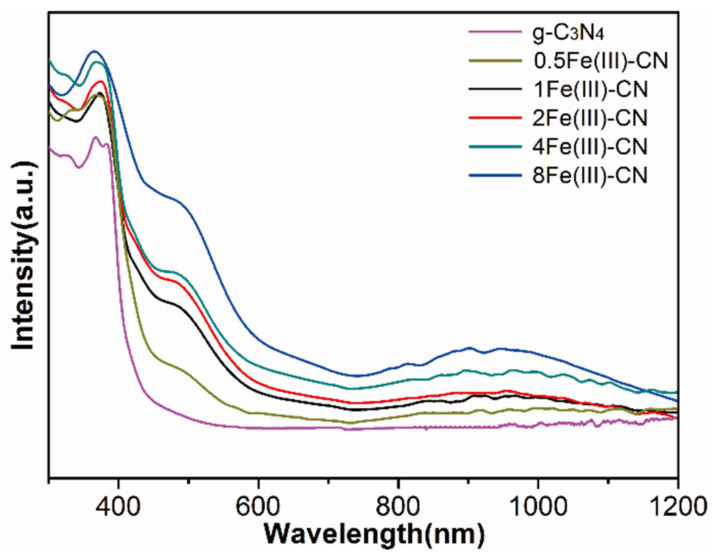
UV-Vis diffuse reflectance absorption spectra of all samples.

The absorbance of all samples was measured by UV-Vis diffuse reflectance spectroscopy, and the results are shown in Figure 7. The pure g-C_3_N_4_ exhibits an absorption edge at about 450 nm. Interestingly, a slightly red shift of the intrinsic absorption edge is observed for the samples with Fe(Ш) doping, and there is an obvious enhancement in the visible light absorption region between 400–800 nm, showing the potential of photocatalysis using visible light. As the amount of FeCl_3_ increases, the absorption capacity increases gradually, and the absorption edge shifts to the long wavelength. Such a phenomenon is ascribed to the interfacial charge transfer effect; a portion of electrons, excited from the valence band, could be used to reduce the Fe(Ш), and the electron transport distance is greatly shortened. Meanwhile, it can be observed that there is an obvious absorption peak at 800 to 1200 nm, revealing that the optical absorption spectrum could extend to the near-infrared region. The intensity of near-infrared absorption increases with increasing Fe(Ш) content, which is related to the increasing amount of FeOOH nanoparticles in the Fe(Ш)-CN as the amount of FeCl_3_ increases.

### 3.6. PL Measurements

**Figure 8:**
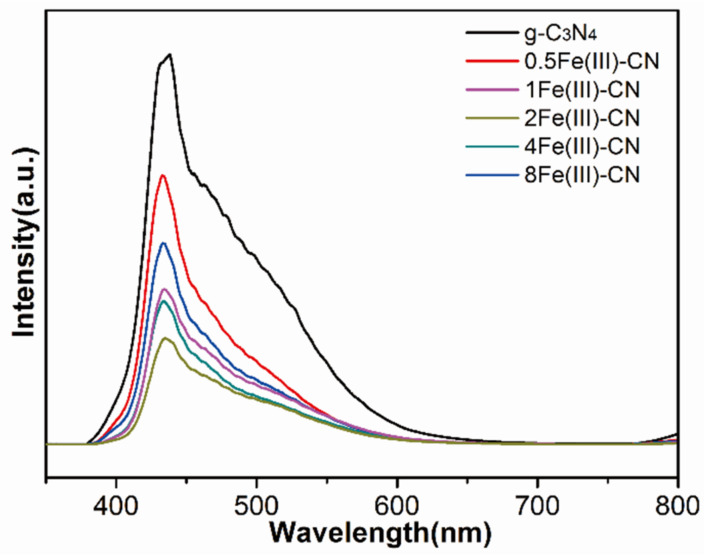
PL spectra of all samples.

The charge separation and transfer characteristics of all samples were investigated by fluorescence spectroscopy. As shown in Figure 8, the g-C_3_N_4_ exhibits the strongest steady-state PL intensity under an excitation wavelength of 325 nm. After Fe(Ш) ions doping, the intensity of all Fe(Ш)-CN samples becomes weakened because Fe(Ш) ions and FeOOH can serve as the electron acceptor, which can inhibit the charge recombination and facilitate the charge transfer. Since superfluous Fe(Ш) ions become recombination center, the PL intensity of Fe(Ш)-doped samples experience the tendency of falling first, and then rising again with increasing feeding amount of FeCl_3_. Specifically, the PL intensity is the weakest with Fe(Ш) ions content of 2%, implying the highest carrier separation efficiency of 2Fe(Ш)-CN.

### 3.7. Electron Spin Resonance Spectra

**Figure 9:**
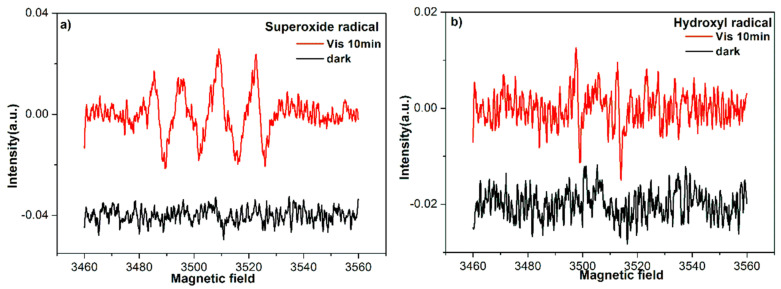
ESR spectra of 2Fe(Ш)-CN in aqueous solution before and after visible light irradiation: (**a**) DMPO- •O_2_^−^ and (**b**) DMPO-•OH.

To confirm the roles of •O_2_^−^ and •OH during photocatalytic process, ESR spectra of 2Fe(Ш)-CN were performed using DMPO as radical trapper under visible light irradiation. As shown in Figure 9, no signals were detected in the dark. After 10 min visible light irradiation, the characteristic signals of the DMPO-•O_2_^−^ (Figure 9a) and DMPO-•OH (Figure 9b) are both clearly observed. The signal of •O_2_^−^ is much stronger than that of •OH under visible light. Because the valance band of g-C_3_N_4_ is at −1.40 eV, it cannot directly oxidize H_2_O or OH^−^ into •OH radicals (E^θ^ (H_2_O/•OH) = 2.40 V, E^θ^ (OH^−^/•OH) = 1.99 V) [35]. Therefore, only a small number of •OH radicals could be generated from the following reaction: •O_2_^−^ + e^−^ + 2H^+^ → H_2_O_2_; H_2_O_2_ + e^−^ → •OH + OH^−^. Thus, it can be seen that •O_2_^−^ is the main active species.

### 3.8. Photocatalytic Activity

**Figure 10:**
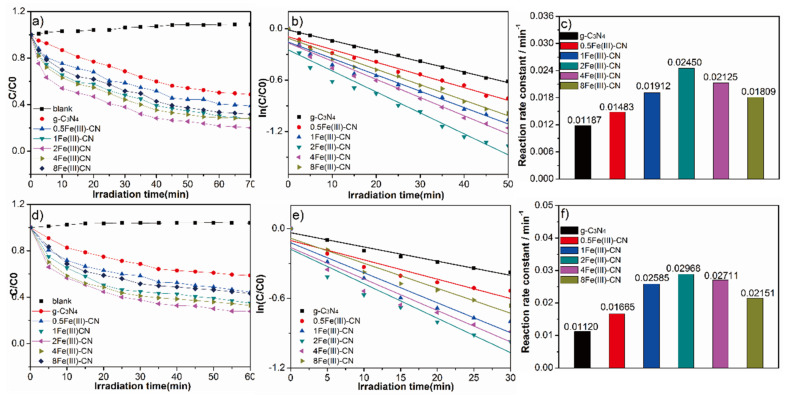
(**a**) Photodegradation rate of MO under visible light irradiation; (**b**) kinetic curves for the photocatalytic degradation of all samples; (**c**) reaction rate constant for the MO degradation; (**d**) photodegradation rate of TC under visible light irradiation; (**e**) kinetic curves for the photocatalytic degradation of all samples; (**f**) reaction rate constant for the TC degradation.

Figure 10 displays the photocatalytic properties of all samples based on the degradation of MO and TC under visible light irradiation. The results indicate that all Fe(Ш)-CN samples exhibit better photocatalytic activities than pure g-C_3_N_4_. In Figure 10a, nearly 80.0% of MO is degraded after 70 min visible light irradiation with 2Fe(Ш)-CN as catalyst, whereas only 51.3% of MO can be degraded with the presence of pure g-C_3_N_4_. The photocatalysis degradation follows the first-order kinetics, expressed as follows: -ln(C/C_0_) = k_app_t. Figure 10b presents the linear relationship between ln(C/C_0_) and time, where C/C_0_ is the normalized MO concentration, t is the reaction time, and k is the reaction rate constant. The corresponding apparent pseudo-first-order rate constant k_app_ of 2Fe(Ш)-CN is 0.02450 min^−1^, which is about 2.06 times higher than that of g-C_3_N_4_, as shown in Figure 10c.

TC is chosen as another different type of model pollutant to evaluate the photocatalytic activity as shown in Figure 10d. TC removal reaches 72.2% during 60 min reaction for the 2Fe(Ш)-CN, but only 41.2% for the pure g-C_3_N_4_. Similarly, Figure 10e presents the linear relationship between ln(C/C_0_) and time, and the corresponding apparent pseudo-first-order rate constant k_app_ of the 2Fe(Ш)-CN is 0.02968 min^−1^, which is about 2.65 times higher than that of g-C_3_N_4_, as shown in Figure 10f. Thus, it can be seen that Fe(Ш) doping is beneficial to improve the photocatalytic activities.

**Figure 11:**
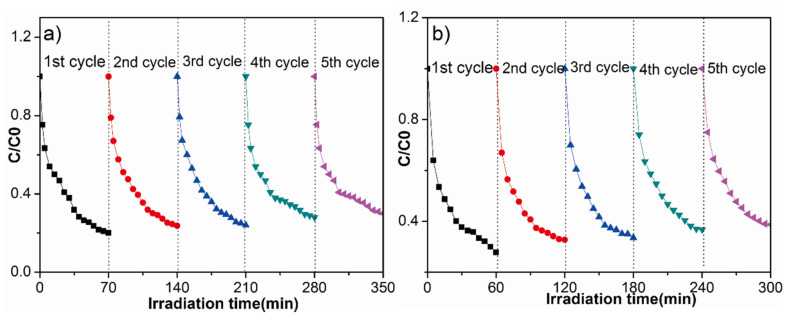
The stability study for the photocatalytic MO (**a**) and TC (**b**) degradation by 2Fe(Ш)-CN.

Besides the photocatalytic performance, the stability of the catalyst is another key factor for its practical applications. Therefore, recycling experiments are carried out on the 2Fe(Ш)-CN sample under the same conditions. According to the results shown in Figure 11, whether for photodegradation of MO or TC, the sample still exhibits superior activity after five cycles, suggesting the excellent stability of the 2Fe(Ш)-CN sample during the photocatalytic reaction.

### 3.9. Mechanism Discussion

**Figure 12:**
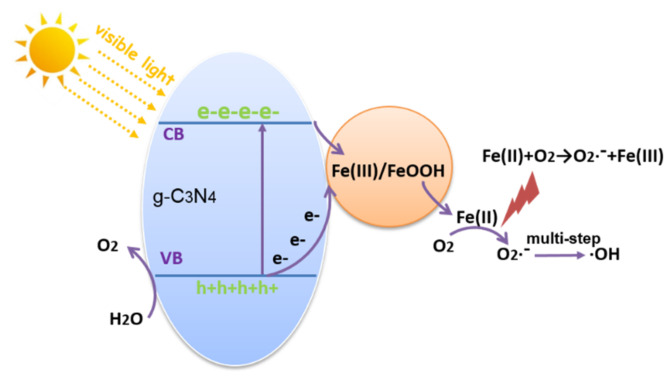
The photocatalytic process of the 2Fe(Ш)-CN.

Compared to the pure g-C_3_N_4_, the photocatalytic performance of all Fe(Ш)-CN samples is much improved, and especially at Fe(Ш) ions content of 2% it exhibits the best photocatalytic activity. Based on the above characterization analysis, the photocatalytic process is shown in Figure 12 and expressed by the following procedures.

g-C_3_N_4_ + hν → h^+^ + e^−^

Fe(Ш)/FeOOH + e^−^ → Fe(II); Fe(II) + O_2_ → •O_2_^−^ + Fe(Ш)

•O_2_^−^ + e^−^ + 2H^+^ → H_2_O_2_; H_2_O_2_ + e^−^ → •OH + OH^−^

•O_2_^−^/•OH/h^+^ + MO/TC → CO_2_ + H_2_O/byproducts

When the Fe(Ш)-CN sample is irradiated by visible light, a part of the electrons of g-C_3_N_4_ are excited from the valence band to the conduction band, leaving holes in the valence band. Another part of the electrons are used to reduce Fe(Ш)/FeOOH to Fe(II) (E(Fe(Ш)/Fe(II)) = 0.77 V), which further reacts with O_2_ to generate •O_2_^−^ directly. In this regard, it promotes the separation of electron-hole pairs. Meanwhile, the transmission distance of electrons is shortened greatly, leading to the enhancement of visible-light absorption ability. The •OH radical is originated from the multi-step reaction with •O_2_^−^. •OH and •O_2_^−^ are both reactive species that can degrade the pollutants.

The doping content of Fe(Ш) ions is an important factor in the activities. When the doping content of Fe(Ш) ions increases from 0 to 2%, the activities improve because Fe(Ш) ions and FeOOH act as electron acceptor. However, with the doping content of Fe(Ш) ions increased continuously, the activities gradually decrease, since Fe(Ш) ions become the recombination center of electron-hole pairs. Meanwhile, the reactive sites are covered with the FeOOH nanoparticles generated on the surface of g-C_3_N_4_.

## 4. Conclusions

In conclusion, Fe(Ш)-CN photocatalysts were prepared using a facile ultrasonic method. All samples exhibited significantly enhanced photocatalytic activities towards the degradation of MO and TC compared to pure g-C_3_N_4_. This is attributed to the enhanced light absorption ability and efficient separation of photogenerated electron-hole pairs due to Fe(Ш) ions doping. The doping content of Fe(Ш) ions is an important factor governing the activities; when the mole percentage ratios of Fe(Ш) ions against g-C_3_N_4_ was 2%, it exhibited the best photocatalytic activities. When the doping content of Fe(Ш) ions increases from 0 to 2%, the activities improve because Fe(Ш) ions and FeOOH act as electron acceptor and promote the separation of electron-hole pairs. Meanwhile, the electron transport distance is sharply shortened and the light absorption capacity increases gradually. However, further increasing Fe(Ш) ions content leads to the decay of activities, since Fe(Ш) ions become the recombination center, and the reactive sites are blocked by the superfluous FeOOH. Moreover, •O_2_^−^ plays an important role during the reaction process.

## Data Availability

Not applicable.
